# Metformin Alleviates Epirubicin-Induced Endothelial Impairment by Restoring Mitochondrial Homeostasis

**DOI:** 10.3390/ijms24010343

**Published:** 2022-12-25

**Authors:** Qi Sun, Huiling Jia, Shuo Cheng, Yujuan Wang, Jun Wang

**Affiliations:** 1 The Center for Ion Beam Bioengineering & Green Agriculture, Hefei Institutes of Physical Science, Chinese Academy of Sciences, Hefei 230031, China; 2 The Science Island Branch of the Graduate School, University of Science and Technology of China, Hefei 230031, China; 3 USTC Life Sciences and Medicine, University of Science and Technology of China, Hefei 230031, China; 4 High Magnetic Field Laboratory of the Chinese Academy of Sciences, Hefei 230031, China

**Keywords:** epirubicin, vascular endothelial injury, metformin, TFAM, mitochondrial dynamics

## Abstract

Vascular endothelial injury is important in anthracycline-induced cardiotoxicity. Anthracyclines seriously damage the mitochondrial function and mitochondrial homeostasis. In this study, we investigated the damage of epirubicin to vascular endothelial cells and the protective role of metformin from the perspective of mitochondrial homeostasis. We found that epirubicin treatment resulted in DNA double-strand breaks (DSB), elevated reactive oxygen species (ROS) production, and excessive Angiotensin II release in HUVEC cells. Pretreatment with metformin significantly mitigated the injuries caused by epirubicin. In addition, inhibited expression of Mitochondrial transcription factor A (TFAM) and increased mitochondria fragmentation were observed in epirubicin-treated cells, which were partially resumed by metformin pretreatment. In epirubicin-treated cells, knockdown of TFAM counteracted the attenuated DSB formation due to metformin pretreatment, and inhibition of mitochondrial fragmentation with Mdivi-1 decreased DSB formation but increased TFAM expression. Furthermore, epirubicin treatment promoted mitochondrial fragmentation by stimulating the expression of Dynamin-1-like protein (DRP1) and inhibiting the expression of Optic atrophy-1(OPA1) and Mitofusin 1(MFN1), which could be partially prevented by metformin. Finally, we found metformin could increase TFAM expression and decrease DRP1 expression in epirubicin-treated HUVEC cells by upregulating the expression of calcineurin/Transcription factor EB (TFEB). Taken together, this study provided evidence that metformin treatment was an effective way to mitigate epirubicin-induced endothelial impairment by maintaining mitochondrial homeostasis.

## 1. Introduction

Despite its well-known cardiotoxicity, the anthracyclines, including epirubicin, idarubicin and doxorubicin (DOX), continue to be extensively available for the treatment of solid tumors and hematologic malignancies, such as breast cancer, esophageal cancer, osteosarcoma, soft tissue sarcomas, lymphomas, leukemias and childhood tumors. The irreversible cardiac damages of anthracyclines, such as cardiomyopathy and heart failure, are cumulative-dose dependent, which in turn affects the prognosis of cancer survivors. The cumulative lifetime doses up to 400 mg/m^2^ of DOX could result in a 5% incidence of congestive heart failure (CHF); When the cumulative lifetime dose reaches 700 mg/m^2^, the incidence of CHF could be as high as 48% [1]. Because of the lack of multicenter randomized controlled clinical studies, the traditional medicines for CHF, such as β-blockers, angiotensin-converting enzyme inhibitors or angiotensin II receptor antagonists, are still in dispute [2,3,4]. Dexrazoxane is the only drug approved by FDA to protect against anthracycline-induced cardiotoxicity, but it also has not shown a definite effect.

Previous studies indicated that anthracyclines damaged cardiomyocytes by inducing excess ROS and inflammation and inhibiting topoisomerase 2β [5,6]. Recently, more attention has been paid to investigating anthracycline-induced vascular endothelium injury and the countermeasures [7,8]. Tao RH et al. demonstrated that vascular damage, which results in decreased cardiac blood flow, also contributed to Dox-induced cardiotoxicity [9]. In addition, DOX-induced endothelial damage has contributed to severe chronic vascular diseases, such as atherosclerosis [10]. In a population-based matched cohort study involving 7289 childhood cancer survivors, even at relatively young ages, survivors still experienced an obviously increased incidence of coronary artery disease compared to the general population (Cause-Specific Hazard Ratio = 3.4, 95% CI) [11]. In the Markus Räsänen et al.’s study, endothelial protection therapy with vascular endothelial growth factor-B(VEGF-B) could inhibit cardiac atrophy and capillary rarefaction of myocardial, and improve endothelial function in DOX-treated Mice, thus exert protection against DOX-induced cardiotoxicity [12]. In addition, another clinical study found that thrombomodulin, a serum biomarker of endothelial dysfunction, was a candidate for predicting the risk of early sub-clinical DOX-induced cardiotoxicity in patients with breast cancer [13].

In this study, compared with patients with normal left ventricular ejection fraction (LVEF), patients with significantly reduced LVEF (>10%) had a higher serum level of thrombomodulin both before and after the first dose of DOX. Epirubicin, an analogue of DOX, is widely used, particularly in patients with established cardiovascular risk factors or a history of anthracyclines use because of comparable antitumor efficacy and lower risk of cardiotoxicity [14]. In a randomized clinical study involving 172 patients with soft-tissue sarcoma, after a median follow-up of 27.7 months, there was an obviously increased incidence of clinically manifest cardiomyopathy in DOX-treated patients (108 cases) receiving a median cumulative dose of 240 mg/m^2^ than that in epirubicin-treated patients (60 cases) receiving a median cumulative dose of 450 mg/m^2^ (0.9% vs. 0%). [15]. One reported side effect of epirubicin was that it led to endothelial cell injury through the oxidative stress-induced activation of p38-MAPK [16]. However, much remains to be done in order to comprehend the mechanisms underlying vascular endothelial injury caused by epirubicin and develop efficient methods for minimizing them.

Anthracyclines specifically bind to phospholipid cardiolipin in the inner mitochondrial membrane where anthracyclines disrupt the electron transport chain (ETC) by inhibiting complexes I and II, then leading to ROS production, thus influencing cellular physiological processes, such as calcium handling, intracellular signaling and apoptosis [17,18,19]. The maintenance of mitochondrial homeostasis is an ideal way to keep the functions of cardiac cells during anthracyclines therapy. Metformin, a widely used antidiabetics, has been found to have cardioprotective effects against anthracycline-induced cardiotoxicity via reducing excessive oxidative stress, inflammation, apoptosis, and improving mitochondrial functions in cultured cells and animal models [20,21,22]. In this study, we investigated the damage mechanism of epirubicin from the perspective of mitochondrial homeostasis and found that metformin could alleviate epirubicin-induced endothelial impairment by restoring mitochondrial homeostasis via the calcineurin/TFEB pathway. The study further expanded the protective effect of metformin and provided new evidence for preventing anthracyclines toxicity.

## 2. Results

### 2.1. Metformin Alleviates Epirubicin-Induced HUVEC Cells Dysfunction

**Figure 1:**
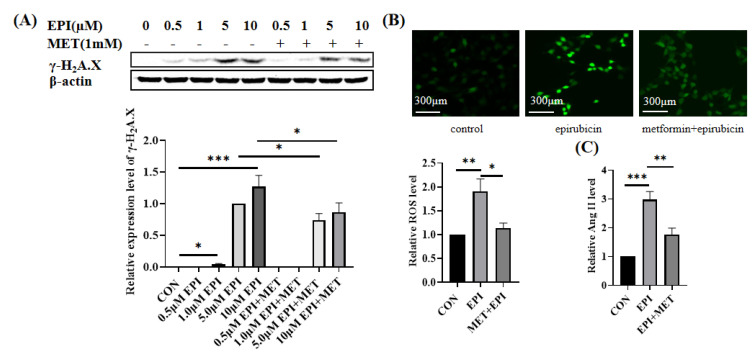
Metformin alleviates epirubicininduced HUVEC cell dysfunction. (**A**) Expression levels of γ−H2A.X. HUVEC cells were respectively incubated with 0.5 μM, 1 μM, 5 μM or 10 μM EPI for 12 h with or without the pretreatment of 1 mM metformin for 12 h. The 5 µM epirubicin treatment group was used as the reference. (**B**) Levels of ROS. HUVEC cells were treated with 5 μM epirubicin for 6 h with or without the pretreatment of 1 mM metformin for 12 h. ROS levels were then detected by 5 mM DCFH−DA probe. (**C**) Levels of Ang II in the supernatant. HUVEC cells were treated with 5 μM epirubicin for 24 h with or without the pretreatment of 1 mM metformin for 12 h. Then, the levels of Ang II in the supernatant were detected by ELISA. EPI—epirubicin, MET—metformin, ns—not significant, * *p* < 0.05, ** *p* < 0.01, *** *p* < 0.001.

Epirubicin is a cell-permeable topoisomerase Ⅱ inhibitor. Its treatment resulted in DNA double-strand breaks in a concentration-dependent manner in HUVEC cells (Figure 1A). If the HUVEC cells were pretreated with 1 mM metformin for 12 hours before epirubicin treatment, attenuated γ-H2A.X levels were observed (Figure 1A). This indicated that metformin can mitigate epirubicin-induced DNA damage. Next, the levels of ROS in 5 μM epirubicin-treated HUVEC cells were detected (Figure 1B). Epirubicin enhanced ROS production by around 89%, and metformin pretreatment notably reversed the ROS accumulation. Angiotensin II (Ang II), an important member of renin-angiotensin-aldosterone system, contributes to many cardiovascular diseases, such as cardiomyopathy and heart failure, hypertension, atherosclerosis and arrhythmia. The elevated release of Ang II reflects dysfunction of cardiovascular endothelia. The levels of Ang II in the supernatant of epirubicin-treated HUVEC cells were measured by ELISA. As shown in Figure 1C, 5 μM epirubicin treatment increased the release of Ang II around two folds. Consistent with the tendencies of DNA damage and ROS level, pretreatment with metformin reduced epirubicin-induced Ang II releasement to a level 70% higher than that of the control cells. Together, these results indicated that metformin protected HUVEC cells against the damage and improved their functions under epirubicin treatment.

### 2.2. Metformin Restores Mitochondrial Biogenesis

**Figure 2:**
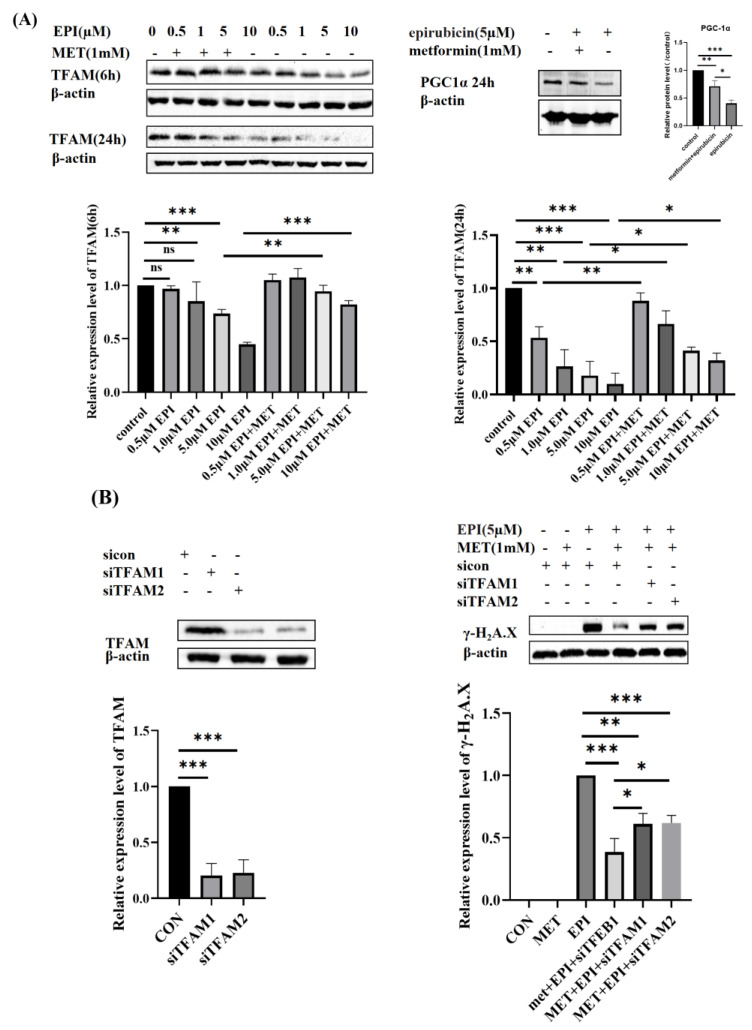
Metformin restores mitochondrial biogenesis. (**A**) Expression levels of TFAM and PGC1α. HUVEC cells were treated with incubated with 0.5 μM, 1 μM, 5 μM or 10 μM EPI, respectively, for 6 h or 24 h with or without the pretreatment of metformin (1 mM) for 12 h. TFAM expression was detected by Western blotting. HUVEC cells were treated with 5 μM epirubicin for 24 h with or without the pretreatment of 1 mM metformin for 12 h, then PGC1α expression was detected by Western blotting. (**B**) Expression levels of γ−H2A.X. HUVEC cells were incubated with TFAM siRNA or control siRNA for 48 h. HUVEC cells were treated with 5 μM epirubicin for 24 h with or without the pretreatment of 1 mM metformin for 12 h. The levels of γ−H2A.X were detected by Western blotting. The epirubicin treatment group was used as the reference. EPI—epirubicin, MET—metformin, ns—not significant, * *p* < 0.05, ** *p* < 0.01, *** *p* < 0.001.

TFAM, an important regulator of mitochondrial biogenesis, plays a key role in the process of replication, transcription and repair of mitochondrial DNA (mtDNA). Since DOX accumulates in mitochondria, we next investigated whether metformin protected HUVEC cells via regulating mitochondrial homeostasis under epirubicin treatment. As shown in Figure 2A, with the increase of epirubicin concentration (0.5 μM, 1 μM, 5 μM, 10 μM), the expression levels of TFAM decreased gradually. At the same time, it was observed that the longer treatment time of epirubicin resulted in lowered expression levels of TFAM. Peroxisome proliferator-activated receptor-γ coactivator1a (PCG-1α) is another major player in mitochondrial biogenesis, which was also inhibited by epirubicin. Pretreatment with 1 mM metformin mitigated the downregulation of TFAM and PCG-1α expression (Figure 2A). To find out how TFAM protected HUVEC against DOX, we inhibited the expression of TFAM in HUVEC cells by small interfering RNA (siRNA) and then treated the cells with epirubicin. As displayed in Figure 2B, metformin mitigated epirubicin-induced DNA damage. However, this was partially reversed by the knockdown of TFAM. These results showed that metformin exerted cardioprotection by promoting TFAM expression.

### 2.3. Metformin Restores Mitochondrial Dynamics

**Figure 3:**
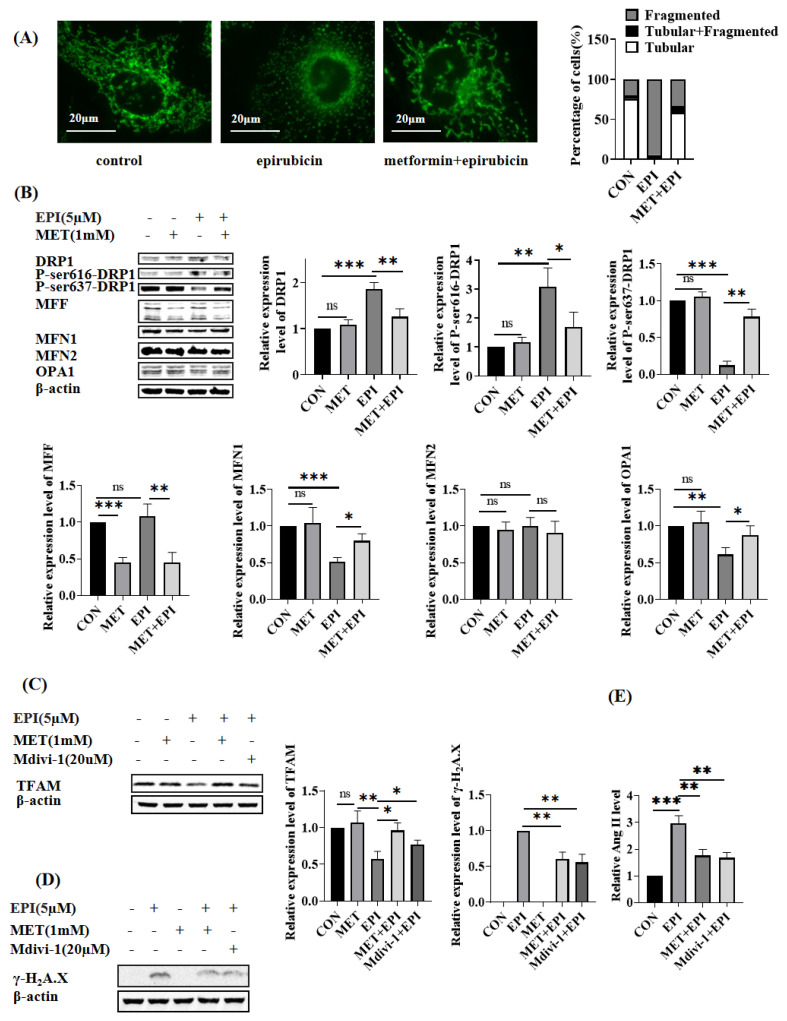
Metformin restores mitochondrial dynamics. (**A**) Mitochondrial morphology analysis by MitoTracker Green. HUVEC cells were treated with 5 μM epirubicin for 24 h with or without the pretreatment of 1 mM metformin for 12 h. Then, the cells were stained with 100 nM MitoTracker Green at 37 °C in the dark for 30 min and imaged. (**B**) HUVEC cells were treated with 5 μM epirubicin for 24 h with or without the pretreatment of 1 mM metformin for 12 h. The expression levels of mitochondrial dynamics related proteins were detected by Western blotting. (**C**). HUVEC cells were treated with 5 μM epirubicin for 12 h with the pretreatment of 1 mM metformin for 12 h or 20 μM mdivi−1 for 2 h, respectively. The expression levels of TFAM were detected by Western blotting. (**D**). HUVEC cells were treated with 5 μM epirubicin for 24 h with the pretreatment of 1 mM metformin for 12 h or 20 μM mdivi−1 for 2 h, respectively. The levels of γ−H2A.X were detected by Western blotting. The epirubicin treatment group was used as the reference. (**E**). HUVEC cells were treated with 5 μM epirubicin for 24 h with the pretreatment of 1 mM metformin for 12 h or 20 μM mdivi−1 for 2 h, respectively. The levels of Ang II in the supernatant were measured by ELISA. EPI—epirubicin, MET—metformin, ns—not significant, * *p* < 0.05, ** *p* < 0.01, *** *p* < 0.001.

Mitochondrial morphology changes closely associate with mitochondrial functions. As shown in Figure 3A, mitochondria in HUVEC cells showed elongated tubules, connecting each other to form networks. By comparison, if the cells were treated with 5 μM epirubicin for 24 h, the mitochondria became dots or short rods without any connection between each other, indicating that epirubicin resulted in mitochondrial fragmentation. In the control group, around 75.5% of HUVEC cells showed tubular mitochondria, and 20.1% of HUVEC cells showed fragmented mitochondria. In the epirubicin-treated HUVEC cells, 3.8% of HUVEC cells showed tubular mitochondria, and 94.6% of HUVEC cells showed fragmented mitochondria. However, metformin pretreatment alleviated epirubicin-induced mitochondrial fragmentation. Percentages of 58.9% and 33.7% of the HUVEC cells in this group bore tubular mitochondria and fragmented mitochondria, respectively.

Mitochondrial morphology is regulated by mitochondrial fusion- or fission-related proteins. DRP1 is involved in mitochondria fission. As shown in Figure 3B, the level of Ser616-phosphorylated DRP1, which promoted mitochondrial fission, was upregulated in epirubicin-treated cells, and the level of Ser637-phosphorylated DRP1, which inhibited mitochondrial fission, was downregulated. In addition, dynamin-like 120 kDa protein, OPA1, MFN1 and Mitofusin 2 (MFN2) are key regulators of mitochondrial fusion. It was observed in Figure 3B that the protein expression levels of MFN1 and OPA1 were repressed by epirubicin treatment, while the expression of MFN2 was not affected. Meanwhile, if HUVEC cells were pretreated with metformin before epirubicin treatment, the changes in the expression of Ser616-DRP1, Ser637-DRP1, OPA1 and MFN1 were partially reversed.

Then. we used Mdivi-1, a specific inhibitor of DRP1, to prevent HUVEC cells from mitochondrial fragments. It was found that the decreased expression of TFAM caused by epirubicin was attenuated (Figure 3C), and the DNA damage caused by epirubicin was also mitigated by Mdivi-1 (Figure 3D). The level of released Ang II in epirubicin-treated cells was consistently decreased by Mdivi-1 pretreatment (Figure 3E). Taken together, epirubicin damaged HUVEC cells, resulting in excessive mitochondrial fission and downregulation of TFAM. The blockage of mitochondrial fission led to notable mitigation of epirubicin-induced damages.

### 2.4. Metformin Restores Mitochondrial Dynamics via Calcineurin/TFEB Pathway

**Figure 4:**
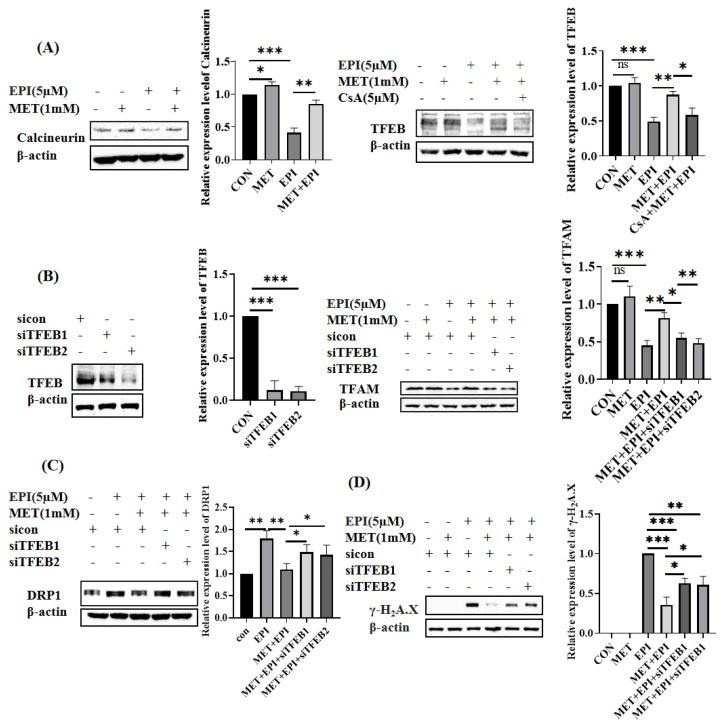
Metformin restores mitochondrial homeostasis via calcineurin/TFEB. (**A**) Metformin restores the calcineurin/TFEB pathway. HUVEC cells were treated with 5 μM epirubicin for 24 h with pretreatment of 1 mM metformin for 12 h or 5 μM CsA for 2 h, respectively. The expression levels of calcineurin and TFEB were detected by Western blotting. (**B**) Expression levels of TFAM. The expression of TFEB in HUVEC cells was inhibited by siRNA. Then, HUVEC cells were treated with 5 μM epirubicin for 12 h with or without the pretreatment of 1 mM metformin for 12 h. Western blotting was used to measure the TFAM expression. (**C**) Expression levels of DRP1. The expression of TFEB in HUVEC cells was inhibited by siRNA. Then, HUVEC cells were treated with 5 μM epirubicin for 24 h with or without the pretreatment of 1 mM metformin for 12 h. DRP1 expression was detected by Western blotting. (**D**) Expression levels of γ−H2A.X. The expression of TFEB in HUVEC cells was inhibited by siRNA. Then, HUVEC cells were treated with 5 μM epirubicin for 24 h with or without the pretreatment of 1 mM metformin for 12 h. Levels of γ−H2A.X were detected by Western blotting. The epirubicin treatment group was used as the reference. EPI—epirubicin, MET—metformin, ns—not significant, * *p* < 0.05, ** *p* < 0.01, *** *p* < 0.001.

TFEB also plays an important role in the maintenance of mitochondrial homeostasis. In our study, the protein levels of TFEB and its upstream regulator calcineurin were downregulated by epirubicin. The metformin pretreatment reversed the epirubicin-induced declines in calcineurin and TFEB protein expression (Figure 4A). In addition, Cyclosporin A(CsA), an inhibitor of calcineurin, alleviated the effect of metformin on the upregulation of TFEB expression. Next, we detected whether TFEB participated in the expression of TFAM after epirubicin and metformin treatment. As shown in Figure 4B, two siRNAs targeted to human TFEB used in this study effectively inhibited the expressions of TFEB. The recovered expression of TFAM in epirubicin-treated HUVEC cells, which were pretreated with metformin, was attenuated by both TFEB siRNAs. In addition, DRP1 expression, which was reduced by metformin in epirubicin-treated HEVEC cells, was upregulated in the condition that TFEB was further knockdown (Figure 4C)**.** Finally, the level of epirubicin-induced DNA damage, which was mitigated by metformin pretreatment, was exacerbated when TFEB expression was knocked down (Figure 4D). Taken together, these results indicated that TFEB is important in regulating the protective role of metformin via maintaining mitochondrial homeostasis and TFAM expression in epirubicin-treated cells.

## 3. Discussion

In recent years, more and more studies have shown that metformin has many functions beyond its hypoglycemic effects, such as suppressing tumor growth, prolonging lifespan, and reducing the incidence of cardiovascular diseases in animal models [23,24,25,26]. In the study, we found metformin could alleviate the epirubicin-induced endothelial injury by decreasing ROS level, DNA damage and the excessive synthesis of Ang II. In addition, we identified metformin exerts an endothelial protective effect by restoring mitochondrial homeostasis via the calcineurin/TFEB pathway.

Oxidative stress plays an important role in DOX-induced cardiotoxicity, and mitochondrial dysfunction, excessive ROS production and reduced expression of the endogenous antioxidant enzyme are all involved in oxidative stress induced by DOX in cardiomyocytes [27,28,29]. Similarly, oxidative stress is also a major reason for anthracycline-induced endothelial injury [16,30], and antioxidant treatment could also alleviate endothelial injury [30] in animal models. In recent years, some clinical studies have also found that metformin has an antioxidant effect. Metformin could decrease the risk of atherogenicity and cardiovascular events in type 2 diabetes (T2D) patients by alleviating oxidative injury in apolipoprotein B100 of LDL [31]. In addition, in coronary artery disease patients without diabetes, metformin could reduce the serum level of thiobarbituric acid reactive substances (TBARs), a marker of oxidative stress [32]. In this study, metformin pretreatment was found to reduce the total cellar ROS in epirubicin-treated HUVEC cells. Pretreatment with metformin could prevent insulin-induced elevated expression of γH2A.X in trophoblast cells [33]. In addition, a clinical study showed that metformin treatment for 3 months (850 mg/day) could reduce γH2A.X expression in the lymphocytes of obese people (body mass index > 30 kg/m^2^) [34]. Consistent with these studies, we found that pretreatment with metformin could alleviate epirubicin-induced γH2A.X in HUVEC cells. Ang II is an important risk factor for many cardiovascular diseases. DOX increased Ang II synthesis in both myocardium and plasma in DOX-treated rats [35,36]. In our study, epirubicin also could increase Ang II synthesis in HUVEC cells which could be alleviated by metformin pretreatment. These results indicated that metformin pretreatment was an effective way to mitigate epirubicin-induced endothelial injury.

TFAM, as a key regulator of mitochondrial DNA (mtDNA) replication, repair and transcription, plays an important role in mitochondrial biogenesis. The study of Zhang D et al. showed that TFAM inactivation induced excessive ROS production, aggravated DNA damage and cardiomyocyte cell cycle arrest, and led to lethal cardiomyopathy [37]. The study of Koh JH et al. showed that besides lowering ROS emissions and oxidative stress, TFAM could increase mitochondrial lipid oxidative capacity and β-oxidative capacity, remodel ETC in a post-translational manner, and attenuate fatty acid-induced membrane depolarization [38]. In the study, TFAM also attenuated insulin resistance by increasing glucose uptake and disposal and enhanced skeletal muscle energy metabolism [38]. In addition, the above-mentioned functions of TFAM were performed through molecular changes rather than a result followed by the replication and transcription of mtDNA [38]. Similar to the result that DOX inhibited TFAM and PGC1α expression in cardiomyocytes [22,39,40], we found that epirubicin suppressed TFAM and PGC1α expression in this study. Previous studies have shown that metformin upregulates TFAM expression in C2C12 myoblasts under hyperglycemia [41] and in the placenta of mice with a high-fat diet [42]. Furthermore, metformin was also found to upregulate myocardial TFAM and PGC1α expression in doxorubicin-treated rats [22]. In our study, the pretreatment with metformin significantly resumed TFAM and PGC1α expression in epirubicin-treated HUVEC cells, as well as decreased the intracellular ROS level, the DNA damage level and the secretion of Ang II. However, in epirubicin-treated HUVEC cells, if TFAM expression was further lowered by siRNA, the effect of metformin on lowering γH2A.X level was attenuated. This indicated that the cardio-protection functions of metformin were in part attributed to its capability to promote TFAM expression and improve mitochondrial biogenesis.

Physiological mitochondrial fission is essential to energy production and impaired mitochondrial clearance. However, excessive mitochondrial fission, also known as mitochondrial fragmentation, can be harmful to endothelial cells. For example, excessive mitochondrial fission induced by silencing MFN1 or MFN2 could damage the mitochondrial function and reduce angiogenic capacity and viability in HUVEC cells [43]. In addition, both in cultured endothelial cells and animal models, excessive mitochondrial fission induced by hyperglycemia could lead to the reduction of nitric oxide (NO) bioavailability, the increase of mitochondria-derived superoxide production, and the induction of apoptosis [44,45,46]. On the contrary, inhibition expression of mitochondrial fission protein 1 (FIS1) or DRP1 could alleviate the hyperglycemia-induced reduction in eNOS activity and NO bioavailability [44]. 

The mitochondrial dynamics are co-regulated by mitochondrial fusion and fission. The separated mitochondria undergo fusion to form an elongated interconnected reticulated structure which keeps electrical and biochemical connectivity and repairs the damaged mitochondria. Therefore, appropriate mitochondrial fusion appears to be beneficial, and inhibition of mitochondrial fusion can impair cell function. Transfection with siRNA against OPA1 significantly increased mitochondrial ROS in Ang II-treated vascular smooth muscle and endothelial cells [47]. Furthermore, the knockdown of MFN1 or MFN2 led to reduced endothelial cell viability and increased apoptosis under low mitogen conditions [43]. Previous studies showed that anthracyclines could induce excessive mitochondrial fission in cardiomyocytes [22,48,49,50], and mitochondrial fission inhibitor (Mdivi-1) and mitochondrial fusion promoter (M1) could protect against DOX-induced cardiotoxicity by inhibiting mitochondrial fission [39,50]. Epirubicin was confirmed to result in excessive mitochondrial fission in HUVEC cells in this study. In addition, mdivi-1 could improve TFAM expression, attenuate DNA damage and reduce Ang II secretion in epirubicin-treated HUVEC cells, revealing that preventing mitochondria from excessive fission could be an effective way to reduce the epirubicin-induced endothelial impairment

We investigated the proteins involved in mitochondrial dynamics to find out the mechanism of epirubicin-induced mitochondrial fission. Inactive DRP1, located in the cytosol, generally makes mitochondrial balance towards fusion. Once DRP1 is activated via phosphorylation under the conditions of stress, FIS1 and MFF recruit DRP1 to the mitochondrial outer membrane where DRP1 GTPase activity makes a multimeric ringlike structure to constrict, finally becoming a pair of separated daughter mitochondria [51]. There is no doubt that phosphorylation at serine 616 of DRP1 promotes mitochondrial fission, and phosphorylation at serine 637 of DRP1 promotes mitochondrial fusion [51]. In our study, compared with the control group, epirubicin slightly increased DRP1 expression and significantly increased DRP1 phosphorylation at Ser616, together with an obvious decrease in DRP1 phosphorylation at Ser637. However, epirubicin did not change MFF expression. Furthermore, epirubicin decreased the expression of mitochondrial fusion-related proteins, such as MFN1 and OPA1, but had no obvious effects on MFN2 expression.

Metformin inhibited high glucose-induced mitochondrial fission in HUVEC cells by reducing DRP1 expression [45], ameliorated Pb-induced mitochondrial fragmentation in SH-SY5Y cells by reducing Drp-1 phosphorylation at the ser616 site [52], inhibited ROS-induced mitochondrial fragmentation by increasing Drp-1 phosphorylation at ser637 site and reducing DRP1 recruitment to mitochondria in adipocytes [53]. In the study of de Marañón AM [54], compared to the control (135 cases), leukocytes from patients with T2D (39 cases) exhibited lower expression of MFN1, MFN2 and OPA1 but higher expression of FIS1 and DRP1, and enhanced leukocyte adhesion to endothelial. Interestingly, in T2D patients (81 cases) with metformin treatment (1700 mg/day for at least 1 year), excessive mitochondrial fission of leukocytes and enhanced leukocyte adhesion could be alleviated. In our study, metformin decreased epirubicin-induced DRP1 upregulation, inhibited DRP1 phosphorylation at the ser616 site, and enhanced DRP1 phosphorylation at the ser637 site in HUVEC cells. In addition, metformin pretreatment could reverse the epirubicin-induced decline of MFN1 and OPA1 expression. Our results differed slightly from Apiwan Arinno‘s study in which metformin could upregulate the expression of MFN1, MFN2 and OPA1 and decrease phosphorylation DRP1 at Ser616 in the myocardium of DOX-treated rats, but neither metformin nor DOX had obvious effects on DRP1 expression [22].

TFEB is a master regulator of the autophagy/lysosomal pathway, which could be activated when exposed to all kinds of stresses, such as mitochondrial stress, oxidative stress, inflammation, endoplasmic reticulum (ER) stress, and pathogen or drug exposure. In recent years, increasing numbers of studies have demonstrated that TFEB could regulate mitochondrial homeostasis in addition to its functions in regulating lysosome biogenesis/autophagy and immune responses [55,56,57,58]. TFEB regulates vascular and vascular endothelial cell functions in a variety of ways. TFEB overexpression inhibited endothelial cell inflammation and attenuated endothelial oxidative injury both in vivo and in vitro [59,60,61], whereas TFEB knockdown aggravated inflammation [59]. In addition, endothelial TFEB can positively regulate angiogenesis and vascular development in animal models by improving the tube formation, proliferation and migration ability of vascular endothelial cells [62,63].

Limited research shows DOX inhibited TFEB expression in cardiomyocytes, and overproduction and/or activation of TFEB in cardiomyocytes prevented DOX-induced ROS overproduction and inflammation [64,65]. Similarly, in our study, epirubicin also could decrease endothelial TFEB expression, which could be reversed by metformin pretreatment. However, the capacity of metformin to remodel mitochondrial homeostasis and to reduce endothelial damage was counteracted partly in epirubicin-treated HUVEC cells with TFEB knockdown, suggesting metformin could exert a protective role by upregulating TFEB expression in epirubicin-treated HUVEC cells. Likewise, in the study of Mansueto G et al., the overexpression of TFEB enhanced mitochondrial biogenesis in mouse skeletal muscle and was accompanied by increased expression of PGC-1α, nuclear respiratory factors 1 (NRF1), nuclear respiratory factors 2 (NRF2), and TFAM [66]. In contrast, the knockout TFEB with siRNA abrogated the increased mRNA expression of PGC1α, NRF1, and TFAM in primary hepatocytes treated with CO-releasing molecules CORM2 [67]. Overexpression of TFEB mitigated excessive mitochondrial fission by decreasing the mRNAs expression of DRP1 and Fis1, thus, reducing cardiomyocyte necrosis and cardiac dilatation in MAO-A Tg mice. [68]. Calcineurin regulates the activity and expression of TFEB [67,69,70]. Even though the mechanisms have not been fully studied, metformin was found to increase the activity [71] and expression of calcineurin [72]. Consistently, our study found that metformin pretreatment reversed the reduction of calcineurin and TFEB expression induced by epirubicin, and the metformin‘s effect on TFEB expression was attenuated by CsA, indicating that metformin could increase TFEB expression via calcineurin in epirubicin-treated HUVEC cells.

In summary, our study indicated that metformin was effective in protecting HUVEC cells against epirubicin-induced injury and disorder of mitochondrial homeostasis. Previous studies have shown that metformin has antitumor effects [73,74] and a synergy effect to enhance the antitumor ability of DOX [75,76,77]. Thus, the application of metformin in tumor patients treated with epirubicin may be beneficial in reducing epirubicin-induced vascular endothelial damage and increasing the antitumor efficacy of epirubicin

## 4. Materials and Methods

### 4.1. Chemicals

The following primary antibodies were utilized: anti-TFAM and anti-β-actin were purchased from Santa Cruz Biotechnology (Dallas, TX, USA), anti-DRP1, anti-P-Ser616-DRP1, anti-P-Ser637-DRP1, anti-Mitochondrial fission factor (MFF), anti-Mitofusin 1 (MFN1), anti-OPA1 and anti-PGC1α were purchased from Cell Signaling Technology (Danvers, MA, USA), anti-TFEB was from ProteinTech (Wuhan, China), anti-calcineurin A was from Abcam, (Shanghai, China). Mitochondrial division inhibitor-1 (Mdivi-1) was purchased from Sigma (Merck KGaA, Darmstadt, Germany). Metformin, epirubicin and Cyclosporin A (CsA) were purchased from MedChemExpress (Monmouth Junction, NJ, USA).

### 4.2. Cell Culture

HUVEC cells line was obtained from the American Type Culture Collection (Manassas, VA, USA) and cultured in Dulbecco’s modified Eagle’s medium/ F12 (Sigma, Merck KGaA, Darmstadt, Germany) supplemented with 10% fetal bovine serum (ShuangRu BioTech, Shanghai, China) and 1% (*v*/*v*) penicillin/streptomycin (Solarbio, Beijing, China) at 37 °C in a humidified 5% CO_2_ atmosphere.

### 4.3. Enzyme-Linked Immunosorbent Assays (ELISA)

Angiotensin II (Ang II) ELISA kit (Elabscience Biotechnology Co., Ltd., Wuhan, China) was used to measure the levels of Angiotensin II released by the cells according to the user’s manual provided by the manufacturer.

### 4.4. Measurement of Cellular Reactive Oxygen Species

Total ROS levels in HUVEC cells were measured using the fluorescence probe DCFH-DA (Molecular Probes, Eugene, OR, USA) according to the manufacturer’s instructions. Cells were mixed with serum-free media containing a 5 mM DCFH-DA probe and incubated at 37 °C in the dark for 30 min. The fluorescent signal intensity was measured with an Olympus IX83 inverted fluorescence microscope equipped with a U-FBNA filter cube (Tokyo, Japan). ImageJ software (National Institutes of Health, Bethesda, MD, USA) was used to analyze the fluorescence intensity. At least 200 cells were analyzed for each sample.

### 4.5. Western Blot

After washing HUVEC cells with cold PBS, the cell lysate was prepared using RIPA buffer containing protease inhibitors cocktail (Roche Diagnostics GmbH, Mannheim, Germany) and protein phosphatase inhibitors cocktail (Sigma, Merck KGaA, Darmstadt, Germany). BCA kit (Sangon Biotech Co., Ltd., Shanghai, China) was used to measure protein concentrations. Based on the molecular weight of the proteins, 8%, 10% or 12% SDS-PAGE were prepared. After transferring onto a polyvinylidene fluoride (PVDF, Roche Diagnostics GmbH, Germany) membrane and incubation in 5% skim milk for 2 h at room temperature, the PVDF membrane was incubated with the primary antibody at 4 °C overnight. Then, the PVDF membrane was washed with TBST (Tris-HCl buffer containing 0.1% Tween-20) three times and incubated with the corresponding HRP-conjugated secondary antibody for two h at room temperature. Protein bands were visualized using a chemiluminescence substrate (Boster, Wuhan, China), and band density was analyzed with ImageJ software.

### 4.6. SiRNA Transfection

The siRNA oligonucleotides targeting human TFAM (siTFAM1: GGACGAAACUCGUUAUCAU; siTFAM2: GGCAAGUUGUCCAAAGAAA) and TFEB (siTFEB1: GCUACAUCAAUCCUGAAAU; siTFEB2: GGCAGAAGAAAGACAAUCA) were synthesized in GenePharma (Shanghai, China) When the cells were grown to 80% confluence in a cell culture dish; the siRNA was mixed with Lipofectamine 2000 (Thermo Fisher, Carlsbad, CA, USA) and loaded into the cell culture. After incubation for 6 h, the medium was refreshed, and the cells were cultured until further treatments and harvest.

### 4.7. Mitochondrial Morphology Analysis

HUVEC cells were seeded and grown on glass coverslips. Mitochondrial morphology was visualized after staining with 100 nM Mito Tracker Green (Keygen Biotech, Nanjing, China) for 30 min and imaged under a fluorescence microscope following the instruction from the manufacturer. Mitochondrial morphologies were divided into three types. “Tubular” meant that over 70% of cellular mitochondria showed tubular morphology. “Fragmented” meant that over 70% of cellular mitochondria showed fragmented morphology. Others were classified as “tubular + fragmented”. At least 300 cells were analyzed for each sample.

### 4.8. Statistical Analysis

All data were presented as mean ± standard deviation from at least three independent experiments performed in triplicate. Statistical significance between the two groups was evaluated using Student’s *t*-test with GraphPad Prism 5 (GraphPad Software, Inc., San Diego, CA, USA). Statistical significance between multiple groups was evaluated using a one-way analysis of variance with SPSS 12.0 software (SPSS, Inc., Chicago, IL, USA). *p* < 0.05 was considered to indicate a statistically significant difference.

## Data Availability

Not applicable.

## References

[1] Swain S.M., Whaley F.S., Ewer M.S. (2003). Congestive heart failure in patients treated with doxorubicin—A retrospective analysis of three trials. Cancer.

[2] Bosch X., Rovira M., Sitges M., Domenech A., Ortiz-Perez J.T., de Caralt T.M., Morales-Ruiz M., Perea R.J., Monzo M., Esteve J. (2013). Enalapril and carvedilol for preventing chemotherapy-induced left ventricular systolic dysfunction in patients with malignant hemopathies: The OVERCOME trial (preventiOn of left Ventricular dysfunction with Enalapril and caRvedilol in patients submitted to intensive ChemOtherapy for the treatment of Malignant hEmopathies). J. Am. Coll. Cardiol..

[3] Heck S.L., Mecinaj A., Ree A.H., Hoffmann P., Schulz-Menger J., Fagerland M.W., Gravdehaug B., Røsjø H., Steine K., Geisler J. (2021). Prevention of Cardiac Dysfunction During Adjuvant Breast Cancer Therapy (PRADA): Extended Follow-Up of a 2 × 2 Factorial, Randomized, Placebo-Controlled, Double-Blind Clinical Trial of Candesartan and Metoprolol. Circulation.

[4] Gujral D.M., Lloyd G., Bhattacharyya S. (2018). Effect of prophylactic betablocker or ACE inhibitor on cardiac dysfunction & heart failure during anthracycline chemotherapy ± trastuzumab. Breast.

[5] Vejpongsa P., Yeh E.T. (2014). Prevention of anthracycline-induced cardiotoxicity: Challenges and opportunities. J. Am. Coll. Cardiol..

[6] Hu C., Zhang X., Zhang N., Wei W.Y., Li L.L., Ma Z.G., Tang Q.Z. (2020). Osteocrin attenuates inflammation, oxidative stress, apoptosis, and cardiac dysfunction in doxorubicin-induced cardiotoxicity. Clin. Transl. Med..

[7] Luu A.Z., Chowdhury B., Al-Omran M., Teoh H., Hess D.A., Verma S. (2018). Role of Endothelium in Doxorubicin-Induced Cardiomyopathy. JACC Basic Transl. Sci..

[8] Sawyer D.B. (2020). Anthracycline-Induced Vascular Dysfunction: Is MitoQ the Answer?. JACC CardioOncol..

[9] Tao R.H., Kobayashi M., Yang Y., Kleinerman E.S. (2021). Exercise Inhibits Doxorubicin-Induced Damage to Cardiac Vessels and Activation of Hippo/YAP-Mediated Apoptosis. Cancers.

[10] Bar-Joseph H., Ben-Aharon I., Tzabari M., Tsarfaty G., Stemmer S.M., Shalgi R. (2011). In vivo bioimaging as a novel strategy to detect doxorubicin-induced damage to gonadal blood vessels. PLoS ONE.

[11] Khanna A., Pequeno P., Gupta S., Thavendiranathan P., Lee D.S., Abdel-Qadir H., Nathan P.C. (2019). Increased Risk of All Cardiovascular Disease Subtypes Among Childhood Cancer Survivors: Population-Based Matched Cohort Study. Circulation.

[12] Rasanen M., Degerman J., Nissinen T.A., Miinalainen I., Kerkela R., Siltanen A., Backman J.T., Mervaala E., Hulmi J.J., Kivela R. (2016). VEGF-B gene therapy inhibits doxorubicin-induced cardiotoxicity by endothelial protection. Proc. Natl. Acad. Sci. USA.

[13] Todorova V.K., Hsu P.C., Wei J.Y., Lopez-Candales A., Chen J.Z.N., Su L.J., Makhoul I. (2020). Biomarkers of inflammation, hypercoagulability and endothelial injury predict early asymptomatic doxorubicin-induced cardiotoxicity in breast cancer patients. Am. J. Cancer Res..

[14] Zamorano J.L., Lancellotti P., Rodriguez Munoz D., Aboyans V., Asteggiano R., Galderisi M., Habib G., Lenihan D.J., Lip G.Y.H., Lyon A.R. (2016). 2016 ESC Position Paper on cancer treatments and cardiovascular toxicity developed under the auspices of the ESC Committee for Practice Guidelines: The Task Force for cancer treatments and cardiovascular toxicity of the European Society of Cardiology (ESC). Eur. Heart J..

[15] Stohr W., Paulides M., Brecht I., Kremers A., Treuner J., Langer T., Beck J.D. (2006). Comparison of epirubicin and doxorubicin cardiotoxicity in children and adolescents treated within the German Cooperative Soft Tissue Sarcoma Study (CWS). J. Cancer Res. Clin..

[16] Yamada T., Egashira N., Bando A., Nishime Y., Tonogai Y., Imuta M., Yano T., Oishi R. (2012). Activation of p38 MAPK by oxidative stress underlying epirubicin-induced vascular endothelial cell injury. Free Radic Biol. Med..

[17] Sarvazyan N. (1996). Visualization of doxorubicin-induced oxidative stress in isolated cardiac myocytes. Am. J. Physiol. Heart C.

[18] Xiong Y., Liu X., Lee C.P., Chua B.H., Ho Y.S. (2006). Attenuation of doxorubicin-induced contractile and mitochondrial dysfunction in mouse heart by cellular glutathione peroxidase. Free Radic Biol. Med..

[19] Yen H.C., Oberley T.D., Gairola C.G., Szweda L.I., St Clair D.K. (1999). Manganese superoxide dismutase protects mitochondrial complex I against adriamycin-induced cardiomyopathy in transgenic mice. Arch. Biochem. Biophys..

[20] Kelleni M.T., Amin E.F., Abdelrahman A.M. (2015). Effect of Metformin and Sitagliptin on Doxorubicin-Induced Cardiotoxicity in Rats: Impact of Oxidative Stress, Inflammation, and Apoptosis. J. Toxicol..

[21] Asensio-Lopez M.C., Lax A., Pascual-Figal D.A., Valdes M., Sanchez-Mas J. (2011). Metformin protects against doxorubicin-induced cardiotoxicity: Involvement of the adiponectin cardiac system. Free Radic Biol. Med..

[22] Arinno A., Maneechote C., Khuanjing T., Ongnok B., Prathumsap N., Chunchai T., Arunsak B., Kerdphoo S., Shinlapawittayatorn K., Chattipakorn S.C. (2021). Cardioprotective effects of melatonin and metformin against doxorubicin-induced cardiotoxicity in rats are through preserving mitochondrial function and dynamics. Biochem. Pharmacol..

[23] Gao J., Yuan J., Wang Q., Lei T., Shen X., Cui B., Zhang F., Ding W., Lu Z. (2020). Metformin protects against PM2.5-induced lung injury and cardiac dysfunction independent of AMP-activated protein kinase alpha2. Redox Biol..

[24] Soberanes S., Misharin A.V., Jairaman A., Morales-Nebreda L., McQuattie-Pimentel A.C., Cho T., Hamanaka R.B., Meliton A.Y., Reyfman P.A., Walter J.M. (2019). Metformin Targets Mitochondrial Electron Transport to Reduce Air-Pollution-Induced Thrombosis. Cell Metab..

[25] Martin-Montalvo A., Mercken E.M., Mitchell S.J., Palacios H.H., Mote P.L., Scheibye-Knudsen M., Gomes A.P., Ward T.M., Minor R.K., Blouin M.J. (2013). Metformin improves healthspan and lifespan in mice. Nat. Commun..

[26] Maruthur N.M., Tseng E., Hutfless S., Wilson L.M., Suarez-Cuervo C., Berger Z., Chu Y., Iyoha E., Segal J.B., Bolen S. (2016). Diabetes Medications as Monotherapy or Metformin-Based Combination Therapy for Type 2 Diabetes: A Systematic Review and Meta-analysis. Ann. Intern. Med..

[27] Wallace K.B. (2007). Adriamycin-induced interference with cardiac mitochondrial calcium homeostasis. Cardiovasc. Toxicol..

[28] Chaiswing L., Cole M.P., Ittarat W., Szweda L.I., St Clair D.K., Oberley T.D. (2005). Manganese superoxide dismutase and inducible nitric oxide synthase modify early oxidative events in acute Adriamycin-induced mitochondrial toxicity. Mol. Cancer Ther..

[29] Zhou S.Y., Starkov A., Froberg M.K., Leino R.L., Wallace K.B. (2001). Cumulative and irreversible cardiac mitochondrial dysfunction induced by doxorubicin. Cancer Res..

[30] Clayton Z.S., Brunt V.E., Hutton D.A., Van Dongen N.S., D’Alessandro A., Reisz J.A., Ziemba B.P., Seals D.R. (2020). Doxorubicin-Induced Oxidative Stress and Endothelial Dysfunction in Conduit Arteries Is Prevented by Mitochondrial-Specific Antioxidant Treatment. JACC CardioOncol..

[31] Rabbani N., Chittari M.V., Bodmer C.W., Zehnder D., Ceriello A., Thornalley P.J. (2010). Increased glycation and oxidative damage to apolipoprotein B100 of LDL cholesterol in patients with type 2 diabetes and effect of metformin. Diabetes.

[32] Mohan M., Al-Talabany S., McKinnie A., Mordi I.R., Singh J.S.S., Gandy S.J., Baig F., Hussain M.S., Bhalraam U., Khan F. (2019). A randomized controlled trial of metformin on left ventricular hypertrophy in patients with coronary artery disease without diabetes: The MET-REMODEL trial. Eur. Heart J..

[33] Vega M., Mauro M., Williams Z. (2019). Direct toxicity of insulin on the human placenta and protection by metformin. Fertil. Steril..

[34] Kudabayeva K., Kosmuratova R., Bazargaliyev Y., Sartayeva A., Kereyeva N. (2022). Effects of metformin on lymphocyte DNA damage in obese individuals among Kazakh population. Diabetes Metab. Syndr..

[35] Zhan C., Bai N., Zheng M., Wang Y., Wang Y., Zhang L., Li J., Li G., Zhao H., Liu G. (2021). Tranilast prevents doxorubicin-induced myocardial hypertrophy and angiotensin II synthesis in rats. Life Sci..

[36] Ma H., Kong J., Wang Y.L., Li J.L., Hei N.H., Cao X.R., Yang J.J., Yan W.J., Liang W.J., Dai H.Y. (2017). Angiotensin-converting enzyme 2 overexpression protects against doxorubicin-induced cardiomyopathy by multiple mechanisms in rats. Oncotarget.

[37] Zhang D., Li Y., Heims-Waldron D., Bezzerides V., Guatimosim S., Guo Y., Gu F., Zhou P., Lin Z., Ma Q. (2018). Mitochondrial Cardiomyopathy Caused by Elevated Reactive Oxygen Species and Impaired Cardiomyocyte Proliferation. Circ. Res..

[38] Koh J.H., Johnson M.L., Dasari S., LeBrasseur N.K., Vuckovic I., Henderson G.C., Cooper S.A., Manjunatha S., Ruegsegger G.N., Shulman G.I. (2019). TFAM Enhances Fat Oxidation and Attenuates High-Fat Diet-Induced Insulin Resistance in Skeletal Muscle. Diabetes.

[39] Yin J., Guo J., Zhang Q., Cui L., Zhang L., Zhang T., Zhao J., Li J., Middleton A., Carmichael P.L. (2018). Doxorubicin-induced mitophagy and mitochondrial damage is associated with dysregulation of the PINK1/parkin pathway. Toxicol. In Vitro.

[40] Liu D., Ma Z., Di S., Yang Y., Yang J., Xu L., Reiter R.J., Qiao S., Yuan J. (2018). AMPK/PGC1alpha activation by melatonin attenuates acute doxorubicin cardiotoxicity via alleviating mitochondrial oxidative damage and apoptosis. Free Radic Biol. Med..

[41] Machado I.F., Teodoro J.S., Castela A.C., Palmeira C.M., Rolo A.P. (2021). miR-378a-3p Participates in Metformin’s Mechanism of Action on C2C12 Cells under Hyperglycemia. Int. J. Mol. Sci..

[42] Jiang S., Teague A.M., Tryggestad J.B., Jensen M.E., Chernausek S.D. (2020). Role of metformin in epigenetic regulation of placental mitochondrial biogenesis in maternal diabetes. Sci. Rep..

[43] Lugus J.J., Ngoh G.A., Bachschmid M.M., Walsh K. (2011). Mitofusins are required for angiogenic function and modulate different signaling pathways in cultured endothelial cells. J. Mol. Cell Cardiol..

[44] Shenouda S.M., Widlansky M.E., Chen K., Xu G.Q., Holbrook M., Tabit C.E., Hamburg N.M., Frame A.A., Caiano T.L., Kluge M.A. (2011). Altered Mitochondrial Dynamics Contributes to Endothelial Dysfunction in Diabetes Mellitus. Circulation.

[45] Wang Q.L., Zhang M., Torres G., Wu S.N., Ouyang C.H., Xie Z.L., Zou M.H. (2017). Metformin Suppresses Diabetes-Accelerated Atherosclerosis via the Inhibition of Drp1-Mediated Mitochondrial Fission. Diabetes.

[46] Wang W.J., Wang Y., Long J.Y., Wang J.R., Haudek S.B., Overbeek P., Chang B.H.J., Schumacker P.T., Danesh F.R. (2012). Mitochondrial Fission Triggered by Hyperglycemia Is Mediated by ROCK1 Activation in Podocytes and Endothelial Cells. Cell Metab..

[47] Robert P., Nguyen P.M.C., Richard A., Grenier C., Chevrollier A., Munier M., Grimaud L., Proux C., Champin T., Lelievre E. (2021). Protective role of the mitochondrial fusion protein OPA1 in hypertension. FASEB J..

[48] Li L., Li J.H., Wang Q.L., Zhao X., Yang D.L., Niu L., Yang Y.Z., Zheng X.X., Hu L.M., Li Y.H. (2020). Shenmai Injection Protects Against Doxorubicin-Induced Cardiotoxicity via Maintaining Mitochondrial Homeostasis. Front. Pharmacol..

[49] Catanzaro M.P., Weiner A., Kaminaris A., Li C.R., Cai F., Zhao F.Y., Kobayashi S., Kobayashi T., Huang Y., Sesaki H. (2019). Doxorubicin-induced cardiomyocyte death is mediated by unchecked mitochondrial fission and mitophagy. FASEB J..

[50] Maneechote C., Khuanjing T., Ongnok B., Arinno A., Prathumsap N., Chunchai T., Arunsak B., Nawara W., Chattipakorn S.C., Chattipakorn N. (2022). Promoting mitochondrial fusion in doxorubicin-induced cardiotoxicity: A novel therapeutic target for cardioprotection. Clin. Sci. (Lond.).

[51] Breitzig M.T., Alleyn M.D., Lockey R.F., Kolliputi N. (2018). A mitochondrial delicacy: Dynamin-related protein 1 and mitochondrial dynamics. Am. J. Physiol. Cell Physiol..

[52] Yang L., Li X., Jiang A., Li X., Chang W., Chen J., Ye F. (2020). Metformin alleviates lead-induced mitochondrial fragmentation via AMPK/Nrf2 activation in SH-SY5Y cells. Redox Biol..

[53] Li A.Y., Zhang S.H., Li J., Liu K., Huang F., Liu B.L. (2016). Metformin and resveratrol inhibit Drp1-mediated mitochondrial fission and prevent ER stress-associated NLRP3 inflammasome activation in the adipose tissue of diabetic mice. Mol. Cell Endocrinol..

[54] de Maranon A.M., Canet F., Abad-Jimenez Z., Jover A., Morillas C., Rocha M., Victor V.M. (2021). Does Metformin Modulate Mitochondrial Dynamics and Function in Type 2 Diabetic Patients?. Antioxid Redox Sign..

[55] Martina J.A., Diab H.I., Brady O.A., Puertollano R. (2016). TFEB and TFE3 are novel components of the integrated stress response. Embo J..

[56] Pastore N., Brady O.A., Diab H.I., Martina J.A., Sun L., Huynh T., Lim J.A., Zare H., Raben N., Ballabio A. (2016). TFEB and TFE3 cooperate in the regulation of the innate immune response in activated macrophages. Autophagy.

[57] Ma X.C., Liu H.Y., Murphy J.T., Foyil S.R., Godar R.J., Abuirqeba H., Weinheimer C.J., Barger P.M., Diwan A. (2015). Regulation of the Transcription Factor EB-PGC1 alpha Axis by Beclin-1 Controls Mitochondrial Quality and Cardiomyocyte Death under Stress. Mol. Cell Biol..

[58] Wang S.J., Chen Y.S., Li X.Y., Zhang W.H., Liu Z.J., Wu M., Pan Q.J., Liu H.F. (2020). Emerging role of transcription factor EB in mitochondrial quality control. Biomed. Pharmacother..

[59] Lu H.C., Fan Y.B., Qiao C.Z., Liang W.Y., Hu W.T., Zhu T.Q., Zhang J.F., Chen Y.E. (2017). TFEB inhibits endothelial cell inflammation and reduces atherosclerosis. Sci. Signal..

[60] Song W., Zhang C.L., Gou L., He L., Gong Y.Y., Qu D., Zhao L., Jin N., Chan T.F., Wang L. (2019). Endothelial TFEB (Transcription Factor EB) Restrains IKK (IkappaB Kinase)-p65 Pathway to Attenuate Vascular Inflammation in Diabetic db/db Mice. Arterioscler Thromb. Vasc. Biol..

[61] Zhou X., Yang J.N., Zhou M., Zhang Y., Liu Y., Hou P.F., Zeng X.L., Yi L., Mi M.T. (2019). Resveratrol attenuates endothelial oxidative injury by inducing autophagy via the activation of transcription factor EB. Nutr. Metab..

[62] Doronzo G., Astanina E., Cora D., Chiabotto G., Comunanza V., Noghero A., Neri F., Puliafito A., Primo L., Spampanato C. (2019). TFEB controls vascular development by regulating the proliferation of endothelial cells. EMBO J..

[63] Fan Y., Lu H., Liang W., Garcia-Barrio M.T., Guo Y., Zhang J., Zhu T., Hao Y., Zhang J., Chen Y.E. (2018). Endothelial TFEB (Transcription Factor EB) Positively Regulates Postischemic Angiogenesis. Circ. Res..

[64] Bartlett J.J., Trivedi P.C., Yeung P., Kienesberger P.C., Pulinilkunnil T. (2016). Doxorubicin impairs cardiomyocyte viability by suppressing transcription factor EB expression and disrupting autophagy. Biochem. J..

[65] Wang X., Wang Q., Li W., Zhang Q., Jiang Y., Guo D., Sun X., Lu W., Li C., Wang Y. (2020). TFEB-NF-kappaB inflammatory signaling axis: A novel therapeutic pathway of Dihydrotanshinone I in doxorubicin-induced cardiotoxicity. J. Exp. Clin. Cancer Res..

[66] Mansueto G., Armani A., Viscomi C., D’Orsi L., De Cegli R., Polishchuk E.V., Lamperti C., Di Meo I., Romanello V., Marchet S. (2017). Transcription Factor EB Controls Metabolic Flexibility during Exercise. Cell Metab..

[67] Kim H.J., Joe Y., Rah S.Y., Kim S.K., Park S.U., Park J., Kim J., Ryu J., Cho G.J., Surh Y.J. (2018). Carbon monoxide-induced TFEB nuclear translocation enhances mitophagy/mitochondrial biogenesis in hepatocytes and ameliorates inflammatory liver injury. Cell Death Dis..

[68] Santin Y., Sicard P., Vigneron F., Guilbeau-Frugier C., Dutaur M., Lairez O., Couderc B., Manni D., Korolchuk V.I., Lezoualc’h F. (2016). Oxidative Stress by Monoamine Oxidase-A Impairs Transcription Factor EB Activation and Autophagosome Clearance, Leading to Cardiomyocyte Necrosis and Heart Failure. Antioxid Redox Sign..

[69] Medina D.L., Di Paola S., Peluso I., Armani A., De Stefani D., Venditti R., Montefusco S., Scotto-Rosato A., Prezioso C., Forrester A. (2015). Lysosomal calcium signalling regulates autophagy through calcineurin and TFEB. Nat. Cell Biol..

[70] Pan B., Li J., Parajuli N., Tian Z.W., Wu P.L., Lewno M.T., Zou J.Q., Wang W.J., Bedford L., Mayer R.J. (2020). The Calcineurin-TFEB-p62 Pathway Mediates the Activation of Cardiac Macroautophagy by Proteasomal Malfunction. Circ. Res..

[71] Chowdhury A.R., Zielonka J., Kalyanaraman B., Hartley R.C., Murphy M.P., Avadhani N.G. (2020). Mitochondria-targeted paraquat and metformin mediate ROS production to induce multiple pathways of retrograde signaling: A dose-dependent phenomenon. Redox Biol..

[72] Eraky S.M., Ramadan N.M. (2021). Effects of omega-3 fatty acids and metformin combination on diabetic cardiomyopathy in rats through autophagic pathway. J. Nutr. Biochem..

[73] Kheirandish M., Mahboobi H., Yazdanparast M., Kamal W., Kamal M.A. (2018). Anti-cancer Effects of Metformin: Recent Evidences for its Role in Prevention and Treatment of Cancer. Curr. Drug Metab..

[74] Tang Z., Tang N., Jiang S., Bai Y., Guan C., Zhang W., Fan S., Huang Y., Lin H., Ying Y. (2021). The Chemosensitizing Role of Metformin in Anti-Cancer Therapy. Anticancer Agents Med. Chem..

[75] Coronel-Hernandez J., Salgado-Garcia R., Cantu-De Leon D., Jacobo-Herrera N., Millan-Catalan O., Delgado-Waldo I., Campos-Parra A.D., Rodriguez-Morales M., Delgado-Buenrostro N.L., Perez-Plasencia C. (2021). Combination of Metformin, Sodium Oxamate and Doxorubicin Induces Apoptosis and Autophagy in Colorectal Cancer Cells via Downregulation HIF-1 alpha. Front. Oncol..

[76] Pateliya B., Burade V., Goswami S. (2021). Enhanced antitumor activity of doxorubicin by naringenin and metformin in breast carcinoma: An experimental study. Naunyn Schmiedebergs Arch. Pharmacol..

[77] Li Y., Wang M., Zhi P., You J., Gao J.Q. (2018). Metformin synergistically suppress tumor growth with doxorubicin and reverse drug resistance by inhibiting the expression and function of P-glycoprotein in MCF7/ADR cells and xenograft models. Oncotarget.

